# Quantification of myocardial perfusion based on signal intensity of flow sensitized MRI

**DOI:** 10.1186/1532-429X-14-S1-O48

**Published:** 2012-02-01

**Authors:** Sumeda B Abeykoon, Janaka Wansapura

**Affiliations:** 1 Physics, University of Cincinnati, Cincinnati, OH, USA; 2 Radiology, Cincinnati Children Hospital Research Center, Cincinnati, OH, USA

## Summary

A new method to quantify myocardial perfusion was developed based on slice select (M_S_) and non-select (M_G_) inversion recovery acquisitions at a single inversion time. A modified Bloch equation was solved to obtain an analytical expression for perfusion (P) in terms of ΔM_SG_ = M_S_-M_G_ The average myocardial perfusion of healthy C57BL/6 mice measured using this technique (P=5.7±0.4 ml/g/min) agreed with that measured using traditional techniques and it had a high reproducibility with mean standard deviation of 3.6% between repeated measures. Perfusion maps of ischemia-reperfusion mice showed significantly low perfusion (P=1.6±0.3 ml/g/min) in the infarcted regions compared to that of remote regions (P=4.1±0.3 ml/g/min,p=0.004).

## Background

The arterial spin labeling technique based on the T1 relaxation time of tissue (T1 method) can be used to quantify myocardial perfusion without the use of exogenous contrast materials[1]. However accurate estimation of T1 relaxation times in the heart, especially in mice is difficult and can require long scan time. As an alternative, we developed a method to quantify myocardial perfusion based on the signal intensity(SI method)of flow sensitized MRI.

## Methods

Myocardial tissue was modeled as intra and extra vascular compartments with fast exchange of spins in between them[1]. The flow sensitization was achieved by slice select (M_S_) and non-select (M_G_) inversion recovery acquisitions at a single inversion time. A steady state gradient echo image (Msa) was also acquired to normalize receiver characteristics. A modified Bloch equation was solved for this acquisition scheme to obtain an analytical expression for perfusion (P) in terms of ΔM_SG_ = M_S_-M_G_ as follows:

P=<ΔM_SG_(t).λ/M_sa_(t)>/(2-Exp(-TR/T_1c_)-TI/T_1c_).T1

where T_1c_=relaxation time of blood,λ=spin density ratio and T1=tissue relaxation time. After validating with flow phantoms (data not shown) the SI method was compared with the conventional T1 method in healthy C57BL/6 mice (n=12). A repeated in vivo experiment was carried out to test the reproducibility of the SI method. Finally, quantitative perfusion maps were obtained in a mouse model of ischemia-reperfusion (n=4) in comparison to delayed Gd enhancement. All experiments were performed on a Bruker 7T scanner and gated gradient echo IR-FLASH and IR- Lock Locker sequences were used for SI and T1 methods with TE=1.7ms, FOV=2.5×2.5cm2, matrix size=128×64, slice thickness=2mm and TR (SI method)≈10×RR, TR(T1 method)≈40×RR.

## Results

The mean left ventricular perfusion in mice derived from the SI method (P=5.7±0.4 ml/g/min) agreed with that obtained from the conventional T1 method (P=5.6±0.3 ml/g/min) and that quantified with fluorescent microspheres (P=5.7±0.3 ml/g/min)[2]. SI method had a high reproducibility with mean standard deviation of 3.6% between repeated measures. Perfusion maps of ischemia-reperfusion mice showed (Figure 2) significantly low perfusion (P=1.6±0.3 ml/g/min) in the hyper intense regions of the corresponding delayed enhanced image compared to remote regions (P=4.1±0.3 ml/g/min, p=0.004).

**Figure 1:**
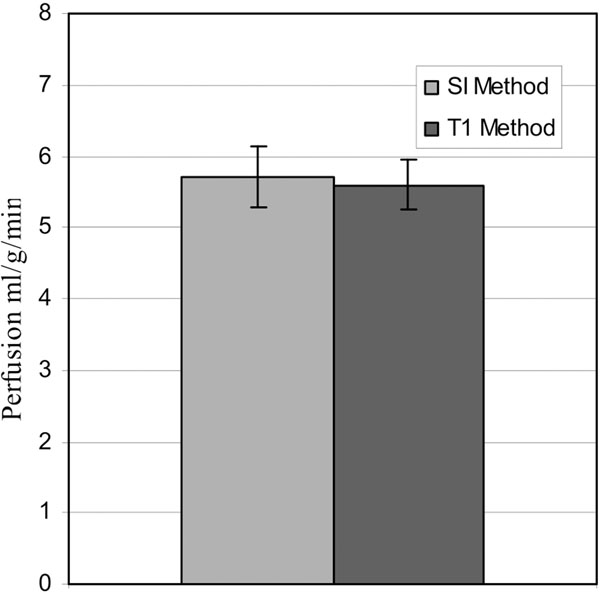
Comparison of SI and T1 methods for myocardial perfusion.

**Figure 2:**
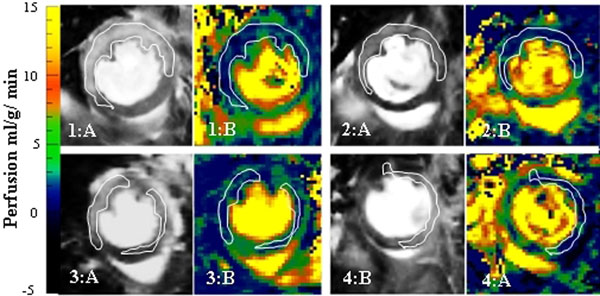
Perfusion deficit regions (right) corresponded well with the myocardial delayed enhancement (left).

## Conclusions

The SI method for perfusion is a robust alternative to the conventional T1 method. In mice it reduces scan time considerably (30%-40%) and is highly reproducible.

## Funding

NIH.
